# Organic food consumption during pregnancy and symptoms of neurodevelopmental disorders at 8 years of age in the offspring: the Norwegian Mother, Father and Child Cohort Study (MoBa)

**DOI:** 10.1186/s12916-024-03685-5

**Published:** 2024-10-21

**Authors:** Johanne T. Instanes, Berit S. Solberg, Liv G. Kvalvik, Kari Klungsøyr, Maj-Britt R. Posserud, Catharina A. Hartman, Jan Haavik

**Affiliations:** 1 Department of Global Public Health and Primary Care, University of Bergen, Bergen, Norway; 2 Department of Biomedicine, University of Bergen, Bergen, Norway; 3 Child- and Adolescent Psychiatric Outpatient Unit, Hospital Betanien, Bergen, Norway; 4 Division of Mental and Physical Health, Norwegian Institute of Public Health, Bergen, Norway; 5 Department of Clinical Medicine, University of Bergen, Bergen, Norway; 6 Division of Psychiatry, Haukeland University Hospital, Bergen, Norway; 7 Gillberg Neuropsychiatry Centre, Institute of Neuroscience and Physiology, the Sahlgrenska Academy, University of Gothenburg, Gothenburg, Sweden; 8 Department of Psychiatry, Interdisciplinary Center Psychopathology and Emotion Regulation, University Medical Center Groningen, University of Groningen, Groningen, The Netherlands; 9 Bergen Center for Brain Plasticity, Division of Psychiatry, Haukeland University Hospital, Bergen, Norway

**Keywords:** Organic food, Prenatal nutrition, Autism spectrum disorder, Attention deficit disorder, Neurodevelopmental disorders, Cohort study, The Norwegian Mother, Father and Child Cohort Study, MoBa, The Medical Birth Registry of Norway, MBRN

## Abstract

**Background:**

Partially driven by public concerns about modern food production practices, organic food has gained popularity among consumers. However, the impact of organic food consumption during pregnancy on offspring health is scarcely studied. We aimed to investigate the association between maternal intake of organic food during pregnancy and symptoms of attention deficit/hyperactivity disorder (ADHD) and autism spectrum disorder (ASD) in offspring at 8 years of age.

**Methods:**

This study was based on the Norwegian Mother, Father and Child Cohort Study (MoBa) and the Medical Birth Registry of Norway (MBRN). The total study sample included 40,707 mother–child pairs (children born 2002–2009). Organic food consumption during pregnancy was assessed by six questions from a food frequency questionnaire in mid-pregnancy (sum score 0–18). Symptoms of ADHD and ASD in the offspring aged 8 years were measured by ADHD (0–54) and ASD (0–39) symptom scores based on the Parent/Teacher Rating Scale for Disruptive Behaviour disorders and the Social Communication Questionnaire. Associations between maternal intake of organic food during pregnancy and symptoms of ADHD and ASD in the offspring were analyzed using regression models with adjustment for covariates such as maternal anxiety and depression, including sibling analysis.

**Results:**

Mean ADHD and ASD symptom scores in the offspring differed only slightly by maternal intake of organic food. The covariate-adjusted unstandardized regression coefficient (adjusted(Adj)beta) with 95% confidence interval for the ADHD symptom score with one unit increase in organic food sum score was 0.03 (0.01, 0.05). Similarly, Adjbeta for autism symptom score was 0.07 (0.04, 0.10). For ADHD, the adjusted estimates weakened when adjusting for maternal symptoms of ADHD. The sibling analyses showed no significant results with Adjbeta − 0.07 (− 0.15, 0.01) and − 0.001 (− 0.12, 0.12) for ADHD and ASD outcomes, respectively.

**Conclusions:**

We observed weak positive associations between frequent maternal organic food consumption during pregnancy and offspring ADHD and ASD symptom levels at 8 years of age. This trend weakened or disappeared after adjusting for maternal symptoms of ADHD, and in sibling analyses, suggesting that the associations mainly reflect genetic confounding. Our study indicates that consumption of organic food during pregnancy should neither be considered a risk factor nor protective against symptoms of ADHD and ASD in offspring.

**Supplementary Information:**

The online version contains supplementary material available at 10.1186/s12916-024-03685-5.

## Background

Neurodevelopmental disorders (NDD), like attention-deficit/hyperactivity disorder (ADHD) and autism spectrum disorder (ASD), are characterized by impaired personal, social, academic, or occupational functioning [1]. The etiology behind NDDs is diverse and complex, and the combination of many different gene variants contributes to the altered neurodevelopment seen in these disorders [2, 3]. Environmental factors are associated with NDDs; among those are well-studied factors related to pregnancy and birth, such as prematurity, low birthweight, maternal obesity, and smoking during pregnancy [2, 4, 5]. Furthermore, although less studied, exercise during pregnancy may favor neurodevelopment in the offspring [6]. However, causal relationships are more difficult to establish, for instance, smoking during pregnancy is associated with increased risk of ADHD and ASD in the offspring, but this relationship may be due genetic factors related to both ADHD/ASD in offspring and maternal smoking behavior, and not the effect by smoking per se [3, 7].

Less is known about the role of nutritional factors during pregnancy on child neurodevelopment. Overall, a modest association has been reported between better maternal diet quality and favorable neurodevelopment in the offspring, such as fewer affective and emotional problems and less hyperactivity [8–11]. More specifically, use of folic acid and multivitamins around the time of conception have been associated with decreased risk of ASD [12, 13]. In studies based on the large Norwegian Mother and Child (MoBa) cohort, higher maternal diet quality during pregnancy was associated with lower child ADHD symptom scores and lower risk for childhood ADHD and ASD diagnosis [14–16]. Conversely, a high intake of ultra-processed food, considered to be an indicator of a lower diet quality, was associated with increased ADHD symptoms [14]. Another study from the same cohort also reported a weak positive association between high maternal intake of sweetened carbonated beverages and child ADHD symptoms at 8 years of age [17].

The consumption of organic food (also known as ecological food or biological food) increases worldwide [18]. Organic farming aims to conserve biodiversity and secure a high standard of animal welfare. The use of synthetic pesticides, chemical fertilizers, genetically modified organisms, and food additives is restricted or prohibited [19, 20]. Organic food is by many consumers perceived as healthier than conventionally produced food, but as available studies are, few there is insufficient evidence to conclude whether organic food is healthier per se [21, 22]. Compared to conventional food, organic food may be more contaminated by pathogenic bacteria [23] but have higher concentrations of antioxidants and omega-3 fatty acids [24, 25]. However, the nutritional significance of these differences has been perceived as marginal [25]. Furthermore, as compared to non-organic food, organic food has been found to contain less pesticide residues and lower concentration of cadmium [24]. Prenatal exposure to pesticides, cadmium, and lead as measured in maternal blood or urine samples has been associated with symptoms of ADHD and ASD in childhood; however, the possible role of organic food intake on these associations has not been addressed [26–28].

There is limited knowledge about possible health implications of organic food consumption during pregnancy. Studies based on the MoBa cohort found that intake of organic food during pregnancy were associated with lower prevalence of hypospadias and unaltered prevalence of cryptorchidism in the child and organic vegetables with decreased risk of pre-eclampsia [29, 30].

The rapid increase of organic food consumption emphasizes the need for more knowledge on possible health consequences of organic food consumption. To the best of our knowledge, intake of organic food during pregnancy has not been studied in the context of symptoms of neurodevelopmental disorders in the offspring.

The object of this study was to investigate the relationship between organic food consumption during pregnancy and symptoms of NDDs in the offspring, focusing on symptoms of ADHD and ASD.

## Methods

### Study design and study sample

The Norwegian Mother, Father and Child Cohort Study (MoBa) is a population-based pregnancy cohort study conducted by the Norwegian Institute of Public Health. Participants were recruited from all over Norway from 1999 to 2008 [30]. The women consented to participation in 41% of the pregnancies. The cohort now includes 114,500 children, 95,200 mothers, and 75,200 fathers. The current study is based on version 12 of the quality-assured data files released for research in January 2019. The participating parents filled out questionnaires prenatally, including information about maternal diet during pregnancy. In addition, questionnaire data was retrieved from the families at several time points, among those collected when the children were 8 years of age. The MoBa study is ongoing. Data from MoBa are routinely linked to the Medical Birth Registry of Norway (MBRN), a national health registry containing information about all births in Norway since 1967 [31].

Although women from all parts of the Norway were invited to participate, MoBa is not fully representative of the total population of Norwegian mothers. Compared to the general population, young mothers (< 25 years), single mothers, non-users of prenatal folic acid supplements, and smokers were, among others, relatively underrepresented in the MoBa cohort [31]. In addition, as all material was written in Norwegian, persons without knowledge of Norwegian could not participate [32]. Furthermore, data was missing from 52% (*n* = 43,828) of the children at 8 years of age in the MoBa cohort [33].

**Fig. 1:**
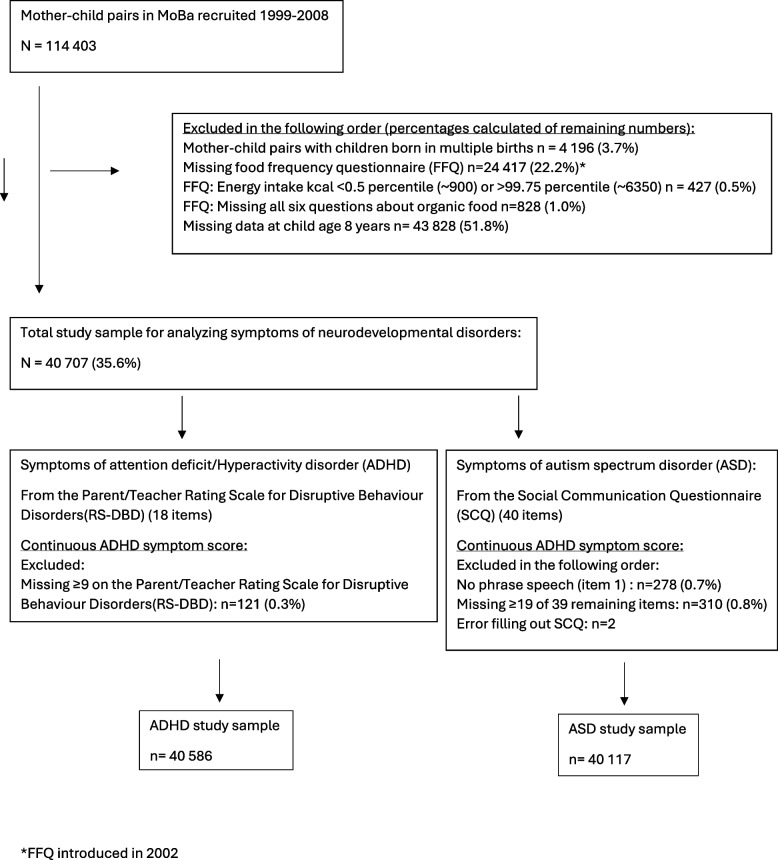
Flowchart of the study population

From the initial sample we had access to 114,403 mother–child pairs with children born 1999–2009, pairs with children born in plural births were excluded (*n* = 4196, 3.7%). Furthermore, pairs were excluded in the following order: missing food frequency questionnaire in mid-pregnancy (*n* = 24,417, 22.2%), calculated daily energy intake < 0.25 percentile (~ 900 kcal) or > 99.75 percentile (~ 6350 kcal) [17] (*n* = 427, 0.5%), or missing all questions about organic food (*n* = 828, 1.0%). Finally, mother–child pairs with missing follow-up data when the child was 8 years were excluded (*n* = 43,828, 51.8%). This resulted in a final study sample of 40,707 (35.6%) mother–child pairs (Fig. 1). The women participating with more than one pregnancy (*n* = 4528) yielded 9206 siblings.

### Exposure

The habitual diet during the first half of pregnancy was assessed by the semi-quantitative MoBa food frequency questionnaire (FFQ) around week 22 of pregnancy. The MoBa FFQ consists of 340 questions organized in 40 groups in accordance with the Norwegian meal pattern [34]. It has been extensively validated and produces a realistic estimate of habitual intake of energy, nutrients and food intake among pregnant women participating in MoBa, as described in detail elsewhere [34–36]. The six questions that cover organic food consumption have not specifically been validated. The mothers were asked if they had used the following organic food products since they became pregnant: milk/dairy, bread/cereal, eggs, vegetables, fruit, and meat, with one question for each food category. The response options were “never or seldom” (0), “sometimes” (1), “often” (2) or “mostly” (3). From this, we calculated a continuous sum score by summarizing values of item scores (0–18) called “sum score of organic food intake” to represent the frequency of maternal organic food intake during pregnancy: from 0 representing “never/seldom” intake of all six food groups to 18 representing “mostly” intake of all six organic food groups. The sum score was calculated by first calculating the mean of all non-missing items and then multiplying by 18, which corresponds to using mean imputation of missing items (1.2%, see Additional file 1 for details). Similar sum score calculations have been used in previous studies [30, 37, 38].

### Outcomes

The mothers filled out questionnaires about symptoms of offspring ADHD and ASD when their children were 8 years old.

#### Symptoms of ADHD

Symptoms of ADHD were assessed by 18 items from the Parent/Teacher Rating Scale for Disruptive Behaviour Disorders (RS-DBD), corresponding to the 9 inattention and 9 hyperactivity/impulsivity symptoms of ADHD [39]. The mothers were asked to score based on what would best describe their children’s behavior the last 6 months. The response options were “never/rarely,” “sometimes,” “often,” or “very often,” given values from 0 to 3.

We used both continuous and categorical measures to represent the number and severity of ADHD symptoms. A “continuous ADHD symptom score” was calculated based on summarizing the values from the RS-DBD sum score (0–54). Questionnaires missing ≥ 9 answers were excluded from analyses (*n* = 121, 0.3%); remaining missing values on individual items (0.3%) were included using mean imputation (Fig. 1, Additional file 1 for details). Furthermore, in supplementary analyses, the scores were categorized as “high” versus "low”; see Additional file 2.

#### Symptoms of ASD

The Social Communication Questionnaire (SCQ) is a 40-item ASD screening tool covering the three symptom domains of ASD (social interaction, communication, repetitive and stereotypic behavior) [40, 41]. Each item is a question with “yes”/”no” response, and the mothers were asked if its content applied to their child or not. The first item was used to evaluate if the child had phrase speech; *n* = 278 (0.7%) reported no phrase speech and were excluded. For the remaining sample, 39 items were scored. Questionnaires missing ≥ 19 of the 39 items were excluded from further analyses (*n* = 310 (0.8%), and *n* = 2 questionnaires were excluded due to error in filling out the questionnaire (Fig. 1). Remaining missing values on individual items (0.7% of total values) were imputed by mean imputation. See Additional file 1 for details.

Each of the 39 items received a value of 0 point for normal behavior and 1 point for abnormal behavior, giving total sum score from 0 to 39. This sum score provided a dimensional measure of ASD symptomatology called “continuous autism symptom score.” Second, in supplementary analyses, scores were categorized as “high” versus “low” ASD symptom scores defined as scoring ≥ 15 and ≤ 14 on the SCQ. See Additional file 2 for details.

#### Covariates

Associations between maternal intake of organic food during pregnancy and symptoms of ADHD and ASD in the offspring were analyzed using regression models with adjustment for covariates.

The selection of covariates was based on previous knowledge regarding organic food consumption during pregnancy in MoBa and on plausible independent risk factors for ADHD and ASD [38, 42–52].

The following covariates were included: birth year, birth season (ADHD only) (January–March, April–June, July–September, October–December), maternal age at delivery (17–45 years), maternal educational level (less than high school, high school, or college/university), parity (nulliparous, 1 previous pregnancy or ≥ 2 previous pregnancies), pre-pregnancy body-mass index (BMI) calculated from self-reported height and weight (kg/m^2^, continuous), smoking during pregnancy (yes/no), alcohol intake during pregnancy (yes/no), and offspring sex (male/female).

Furthermore, maternal total energy intake (kcal/day) and fiber consumption (gram/day) were included as covariates as measures of diet quantity and quality [8, 53]. Energy-adjusted intakes are relevant when focusing on the composition of food, rather than absolute intake [34]. Fiber can be regarded as a marker for a healthy diet and thus a measure of diet quality [8, 53–55]. Energy and fiber intake were calculated based on information from the MoBa FFQ with the use of the Norwegian food composition table and FoodCalc [56, 57].

Additional covariates included symptoms of anxiety, depression, and ADHD in parents. We were not able to adjust for parental symptoms of ASD due to lack of information. Maternal anxiety and depression have been associated with poor maternal diet quality during pregnancy [58–63]. Similar information on symptoms in fathers was of interest, as associations have been found between maternal and paternal diet quality during pregnancy [64]. Both paternal and maternal depression and/or anxiety have been found to increase the risk of ADHD in the offspring [62, 63]. ADHD has been associated with unhealthy dietary patterns and a high heritability has been shown [65–68].

Symptoms of anxiety and depression were assessed by eight items from the Hopkins Symptom Checklist-25(SCL-8) around gestational week 30, where the mother described symptoms of anxiety and depression the last two weeks [69]. We calculated the mean scores based on the response categories “not bothered,” “a little bothered,” “quite bothered,” and “very bothered.” Information on symptoms of ADHD in mothers during the last 6 months was collected from the Adult ADHD Self-Report Scale Screener (ASRS-6) when the child was 3 years of age [70]. The ASRS-6 consists of six items with a 0–4 response scale with the options “never,” “rarely,” “sometimes,” “often,” or “very often.” Summing the responses yielded a sum score with the range 0–24 and a mean score with the range of 1–4. Similar information from SCL-8 was collected from the father around gestational week 15.

Information on covariates was collected from MoBa, with exceptions from maternal age at delivery, birth year, parity, and birth season, which were obtained from MBRN.

### Statistical analyses

**Fig. 2:**
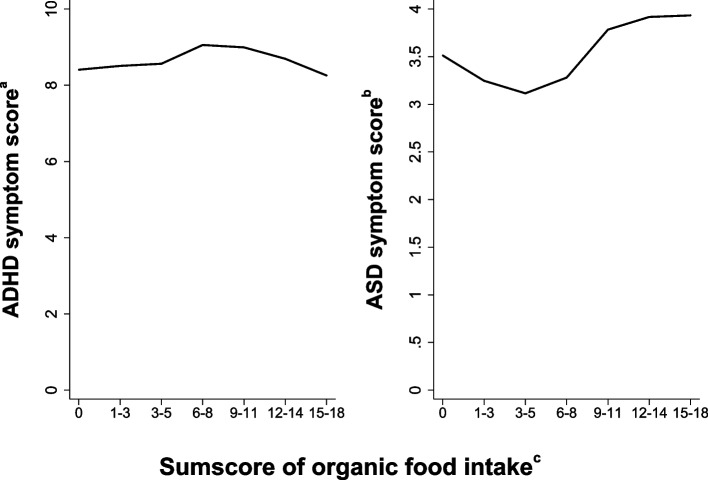
Mean offspring ADHD and ASD symptom scores by sum score of organic food intake (categorized). The “a” symbol indicates the following: offspring symptoms of attention-deficit/hyperactivity disorder (ADHD) at 8 years of age screened by the Parent/Teacher Rating Scale for Disruptive Behaviour Disorders (RS-DBD); continuous ADHD symptom score 0–54. The “b” symbol indicates the following: offspring symptoms of autism spectrum disorder (ASD) at 8 years of age screened by the Social Communication Questionnaire (SCQ); continuous ASD symptom score 0–39. The “c” symbol indicates the following: continuous sum score of organic food intake (0–18) represent the frequency of maternal organic food intake during pregnancy; from 0 representing no/seldom intake to 18 representing frequent. The sum score is categorized into 5 categories

We used linear regression to estimate unstandardized regression coefficients (beta) with 95% confidence intervals (CI) to explore possible associations between organic food intake during pregnancy and ADHD or ASD symptoms scores in offspring. The association between the continuous ASD symptom score and the sum score of organic food intake (total range 0–18) was non-linear (Fig. 2). We therefore stratified the linear regression analyses into two models: organic sum score 0–5 (low organic sum score; 0–5) and 6–18 (high organic sum score; 6–18). Additionally, in a supplementary sensitivity analysis, to capture more extreme groups, we estimated relative risks to evaluate categorized exposure and outcomes; see Additional file 2 for details. For each of the six organic food groups, we used log-binominal regression to compare those eating often/usually of the food group (for instance often/usually eat organic eggs (high organic egg consumption) with those never/seldom eating organic food at all.

Clustered robust standard errors were used to account for women participating with more than one pregnancy, using mother’s ID as cluster variable. Heteroscedasticity was evaluated by plotting residuals against predicted values of the outcomes and found not to be of concern.

The covariates were treated as continuous when the relationship between the covariates and outcomes were linear. With non-linear relationships, the covariates were categorized. Spline modeling was used in a sensitivity analysis (see Additional file 2 for details).

Due to missing data in the variable on maternal symptoms of ADHD (18%), a subsample excluding participants lacking this information were used when adjusting for this variable (*n* = 31,411). Information on paternal symptoms of anxiety, depression, and ADHD were available in a subsample (*n* = 32,806) of the total study population, and this sample was used when these symptoms were included as covariates.

To assess possible sex differences, we refitted the adjusted model with the addition of an interaction term between exposure and offspring sex(male/female) and performed the adjusted model stratified by offspring sex.

Some women participated in MoBa with multiple pregnancies (*n* = 4528), which made it possible to carry out sibling-matched fixed effect models to investigate unmeasured confounding shared by the siblings. Siblings share unobserved variables, such as genetics. Siblings discordant on the exposure (having different values of sum score of organic food intake) contributed to the analyses (*n* = 5534 with ADHD and *n* = 4367/1104 with ASD “low” versus “high” symptom score as outcomes, respectively). By including only those discordant, where the mother used organic food differently in the pregnancies, genetic factors were partly controlled for. The covariates in these analyses were the same as those used in the adjusted model. Due to low statistical power, it was not possible to evaluate categorical outcomes to provide meaningful results.

The analyses were conducted in STATA version 16.1 (College Station, TX) [71].

## Results

### Study population

Table 1, Additional file 3: Tab. S1, and Additional file 4: Tab. S2 show the characteristics of the mother–child pairs in the ADHD and ASD study samples. Mean ADHD and ASD symptom scores in offspring differed only slightly by sum score of organic food intake (Fig. 2).

**Table 1:** Sample characteristics of mother–child pairs

Study sample^a^	ADHD study sample^b^	ASD study sample^c^	
	*n* (%)	*n* (%)	
		“Low” organic sum score (0–5)	“High” organic sum score (6–18)
Mother–child pairs	40,586 (100)	34,421 (100)	5696 (100)
*Birth year*			
2002	2023 (5.0)	1801 (5.2)	204 (3.6)
2003	5783 (14.3)	5118 (14.9)	585 (10.3)
2004	5625 (13.9)	4963 (14.4)	622 (10.9)
2005	6469 (15.9)	5574 (16.2)	803 (14.1)
2006	7264 (17.9)	6189 (18.0)	989 (17.4)
2007	6675 (16.5)	5513 (16.0)	1061 (18.6)
2008	5348 (13.2)	4188 (12.2)	1118 (19.6)
2009	1399 (3.5)	1075 (3.1)	314 (5.5)
*Maternal age at delivery (years)*			
< 17–19	183 (0.5)	122 (0.4)	55 (1.0)
20–24 years	3040 (7.5)	2516 (7.3)	484 (8.5)
25–29 years	12,987 (32.0)	11,214 (32.6)	1645 (28.9)
30–34 years	16,589 (40.9)	14,132 (41.0)	2271 (39.8)
35–39 years	6853 (16.9)	5686 (16.5)	1081 (19.0)
40 years or older	934 (2.3)	751 (2.2)	160 (2.8)
*Maternal age at delivery (years)*			
Mean score (SD)^d^	30.7 (4.4)	30.6 (4.3)	30.8 (4.7)
Missing^e^	19 (0.05)	13 (0.04)	15 (0.04)
*Maternal education*^f^			
Less than high school	645 (1.6)	504 (1.5)	120 (2.1)
High school	11,242 (28.3)	9508 (27.6)	1574 (27.6)
4 years or more college/university	27,844 (70.1)	23,685 (68.8)	3884 (68.2)
*Child ADHD symptom score (continuous)*^g^			
Mean (SD)	8.5 (7.2)	8.5 (7.1)	8.7 (7.4)
*Child autism symptom score (continuous)*^h^			
Mean (SD)	3.4 (2.9)	3.4 (2.9)	3.4 (3.0)

^a^Based on the Norwegian Mother, Father and Child Cohort Study (MoBa)

^b^Attention deficit/hyperactivity disorder (ADHD)

^c^Autism spectrum disorder (ASD). ASD study sample stratified into two: organic sum score 0–5 (“low” organic sum score; 0–5) and 6–18 (“high” organic sum score; 6–18)

^d^SD = standard deviation

^e^Missing due to not specific information about maternal age if age < 17 or > 45

^f^Missing information ADHD sample *n* = 860 (2.1%), ASD sample “low” organic *n* = 724 (2.1%) and “high” organic *n* = 118 (2.1%)

^g^Offspring symptoms of ADHD screened at 8 years of age by the Parent/Teacher Rating Scale for Disruptive Behaviour Disorders (RS-DBD); continuous ADHD symptom score 0–54

^h^Offspring symptoms of ASD screened at 8 years of age by the Social Communication Questionnaire (SCQ); continuous ASD symptom score 0–39

Additional file 5: Tab. S3 shows the number of participants with high consumption of each of the six organic food categories. The percentages of participants defined with high consumption of the six organic food groups varied considerably; 10.2% reported a high consumption of organic eggs, whereas only 3.3% had high consumption of organic meat.

### ADHD

**Fig. 3:**
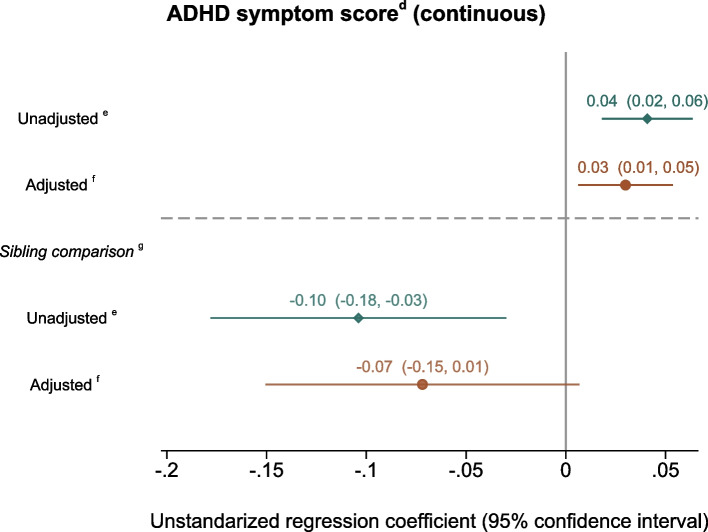
Symptoms of ADHD^a^ in offspring by maternal organic food intake (continuous sum score)^b^ during pregnancy^c^. The “a” symbol indicates the following: offspring symptoms of attention-deficit/hyperactivity disorder (ADHD) screened at 8 years of age by the Parent/Teacher Rating Scale for Disruptive Behaviour Disorders (RS-DBD). The “b” symbol indicates the following: continuous sum score of organic food intake (0–18) represent the frequency of maternal organic food intake during pregnancy; from 0 representing no/seldom intake to 18 representing frequent intake. The “c” symbol indicates the following: data from the Norwegian Mother, Father and Child Cohort Study (MoBa). The “d” symbol indicates the following: continuous ADHD symptom score 0–54 based on information from RS-DBD. The “e” symbol indicates the following: unadjusted linear regression model (*n* = 40,586 ADHD study sample). The “f” symbol indicates the following: adjusted linear regression model (*n* = 37,822 complete cases ADHD study sample). Adjusted for birth year; birth season; maternal age at delivery; maternal educational level; parity, pre-pregnancy body mass index (BMI); maternal smoking, alcohol, energy and fiber intake during pregnancy; maternal symptoms of depression and anxiety in pregnancy measured around gestational week 30. The “g” symbol indicates the following: sibling-matched fixed effect models, siblings discordant for the exposure contribute (*n* = 5534)

We observed a weak positive association between the continuous sum score of organic food intake and the continuous ADHD symptom score, in the adjusted model the unstandardized regression coefficient (adjusted(Aadj)beta) with 95% confidence interval was 0.03 (0.01, 0.05) (Fig. 3). A comparison of the crude and adjusted estimates revealed that adjusting for maternal symptoms of anxiety and depression attenuated risk estimates. The adjusted estimate without this covariate was similar to the crude estimate Adjbeta = 0.04 (0.02, 0.07); thus, inclusion of the other covariates had minimal effects. Including maternal symptoms of ADHD (subsample *n* = 31,411) further weakened the results, i.e., Adjbeta = 0.02 (− 0.008, 0.04). The sibling comparison analysis showed a trend towards a small negative association, with Adjbeta =  − 0.07 (− 0.15, 0.01).

Likewise, supplementary analyses showed that the findings were not significant when assessing exposure and outcome as categorical variables (Additional file 6: Fig. S1).

Including paternal symptoms of anxiety and depression as adjustment variables slightly weakened the results, as compared to the those from the adjusted model in the total sample (data from subsample (*n* = 32,806) (Additional file 7: Fig. S2).

When stratifying the results by offspring sex, males had slightly lower point estimates than females, confidence intervals were overlapping, and there was no statistically significant interaction between sex and organic food intake on ADHD symptom scores (Additional file 8: Fig. S3).

When assessing the organic food groups separately, the results overlapped and there were no significant findings (Additional file 9: Fig. S4).

### ASD

**Fig. 4:**
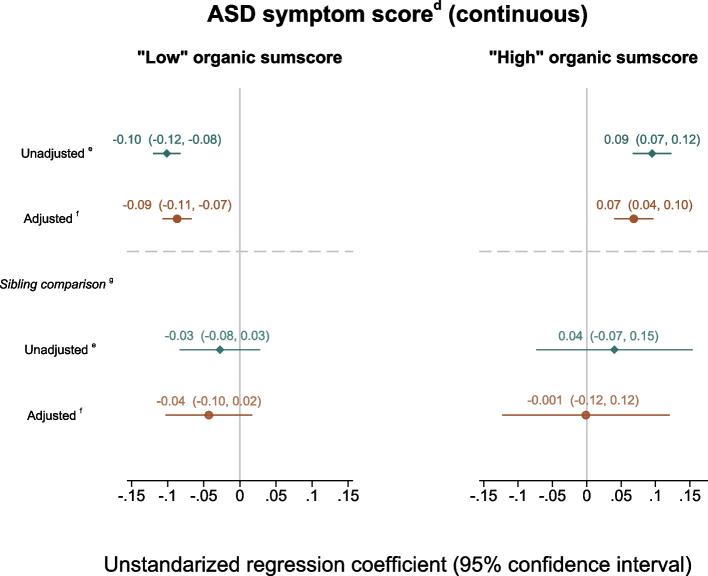
Symptoms of ASD^a^ in offspring by maternal organic food intake (continuous sum score)^b^ during pregnancy^c^. The “a” symbol indicates the following: offspring symptoms of autism spectrum disorder (ASD) screened at 8 years of age by the Social Communication Questionnaire (SCQ). The “b” symbol indicates the following: continuous sum score of organic food intake (0–18) represent the frequency of maternal organic food intake during pregnancy; from 0 representing no/seldom intake to 18 representing frequent intake. The “c” symbol indicates the following: based on the Norwegian Mother, Father and Child Cohort Study (MoBa). The “d” symbol indicates the following: continuous ASD symptom score 0–39 based on information from SCQ. The association between the continuous ASD symptom score and the sum score of organic food intake (total range 0–18) was non-linear. The linear regression analysis was thus stratified into two: “low” organic sum score(0–5) and “high” organic sum score(6–18). The “e” symbol indicates the following: unadjusted linear regression model (ASD study sample *n* = 40,117; “low” organic sum score *n* = 34,421; “high” organic sum score *n* = 5696. The “f” symbol indicates the following: adjusted linear regression model (ASD study sample *n* = 37,394 complete cases; “low” organic sum score *n* = 32,148; “high” organic sum score *n* = 5246). Adjusted for birth year; maternal age at delivery; maternal educational level; parity; pre-pregnancy body mass index (BMI); maternal smoking, alcohol, energy and fiber intake during pregnancy; maternal symptoms of depression and anxiety in pregnancy measured around gestational week 30. The “g” symbol indicates the following: sibling-matched fixed effect models, siblings discordant for the exposure contribute; “low” organic sum score *n* = 4367; “high” organic sum score *n* = 1104

We tested two linear regression models for ASD as outcome. For those with “low” organic sum scores (0–5), there was a small negative association with Adjbeta = − 0.09 (− 0.11; − 0.07) as compared to the “high” group (organic sum score 6–18) which showed a small positive association with Adjbeta = 0.07 (0.04, 0.10) (Fig. 4). However, the sibling comparison analyses showed weaker effects with nonsignificant results (Adjbeta = − 0.04 (− 0.10, 0.02) and − 0.001 (− 0.12, 0.12) for the “low” organic as compared to the “high” organic group, respectively. Likewise, the findings were not significant when assessing exposure and outcome as categorical variables (Additional file 10: Fig. S5).

Including paternal and maternal symptoms of anxiety and depression as covariates had negligible influence on the results (data from subsample *n* = 32445) (Additional file 7: Fig. S2).

When stratifying the results by offspring sex, males had only slightly lower point estimates than females, confidence intervals were overlapping, and there was no statistically significant interaction between sex and organic food intake on ASD symptom scores (Additional file 8: Fig. S3).

There were no significant results when assessing the organic food groups separately; however, there were differences in point estimates ranging from meat with adjusted Relative Risk (AdjRR) = 1.03 (0.40, 2.70) to bread/cereals AdjRR = 1.72 (0.95, 3.10) (Additional fil 9: Fig. S4). Notably, results were unprecise with large confidence intervals, as subgrouping also affects the statistical power of these analyses.

## Discussion

In this large prospective population-based pregnancy cohort study, we observed that frequent consumption of organic food during pregnancy was either weakly or not at all associated with symptoms of ADHD and ASD in the offspring at 8 years of age. An exception was the ASD group with minimal or no exposure of organic food, where we found a weak negative association. None of the associations differed significantly by offspring sex. Adjustment for maternal and paternal symptoms of anxiety, depression, and ADHD slightly weakened the associations. The associations were further attenuated or disappeared in the sibling comparison analyses.

Weak associations have been found between healthy dietary patterns in pregnancy and lower symptoms of ADHD at 8 years of age in the MoBa cohort [14, 15]. A study including data from two US prospective cohorts also found a small positive association between a Western diet and symptoms of ASD in the offspring, but no clear associations were found when investigating dietary patterns considered as healthier than the Western diet, like the Mediterranean diet [72]. The overall organic diet quality has scarcely been studied; findings so far have indicated that organic diet may be considered as healthy, but there is also a concern that such diet may have non-beneficial effects [21, 37].

Compared to what is known about the overall effects of organic diets, more is known about pesticides residues in food. Prenatal exposure to pesticides has been associated with symptoms of ADHD and ASD in childhood [26–28]. For the current study, it was not possible to compare the amount of toxic components, like pesticides, in non-organic versus organic food, but limited findings from other studies have shown that organic food contains fewer synthetic pesticides and pesticide residues as compared to non-organic food [25, 73, 74]. Thus, it is reasonable to expect that mothers with frequent intake of organic food during pregnancy participating in MoBa will consume fewer pesticides as compared with the other participating mothers. We had therefore anticipated a protective effect of frequent organic food consumption during pregnancy on offspring symptoms of ADHD and ASD, in line with these previous findings, yet we could not confirm this hypothesis. In general, Norwegian food is considered nutritious and contain little pesticides residues [74, 75]. Thus, the difference in amount of nutrients and pesticides between organic and non-organic diets in Norway may be too small as to discover possible differences as related to offspring symptoms of ADHD and ASD.

Possible health effects associated with organic diet consumption can be confounded by personal, demographic, and socioeconomic characteristics [38, 73]. As described in detail elsewhere, MoBa participants with frequent intake of organic food were distributed across different socioeconomic groups and not necessarily prone to a generally healthy lifestyle [38]. Furthermore, the diet among frequent organic consumers in MoBa was considered healthier compared to those with no or low organic food intake, with a higher intake of fiber and nutrients such as vitamin C and folate [37]. In addition, frequent organic food intake was related to higher total energy intake, probably related to higher levels of physical activity [37]. We included several covariates in the regression analyses, such as maternal education, maternal alcohol consumption and smoking during pregnancy, and maternal fiber and energy intake, among others. This resulted in only slightly alterations in the risk estimates. We used total fiber intake as a proxy for diet quality, not focusing on specific nutrients. The results indicate that these factors did not greatly impact the associations between organic food intake during pregnancy and offspring symptoms of ADHD and ASD.

Organic eggs were more frequently used compared to other organic food groups, as 10.2% of the mothers were defined as being in the high consumer group, compared to only 3.3% for organic meat. This is in line with Norwegian data from the 2000s, reporting that eggs had the highest organic portion of the total sale in this food group, while meat and fruit were among the lowest [76]. Due to limitations such as small sample sizes in some of the organic food groups, one should be cautious to interpret these results.

Different factors, e.g., socioeconomic status may influence how questionnaires are answered and how children’s symptoms and behaviors are reported. However, we adjusted for maternal education as a proxy for socioeconomic factors, and other factors such as income and employment are highly correlated in MoBa [32]. Symptoms of psychiatric disorders such as anxiety, depression, and ADHD may also influence how participants answer the questionnaires. They may over-report because they are more attentive to symptoms related to these disorders or under-report because they have challenges evaluating the questions. ADHD and ASD outcomes in the present study were based on the mothers’ report of their own children’s symptoms. A history of complications in the pregnancy or illness in the newborn could make the parents more anxious and attentive to their children’s symptoms and therefore more prone to report such symptoms. In our data, the percentage of very preterm birth among offspring defined as high ADHD symptom scorers was 2.5%, compared to 1.1% among those scoring as low (*p* < 0.01). The corresponding percentages were 1.2% among high ASD symptom scorers and 3.8% among those scoring as low (*p* < 0.01). Since our outcomes were not based on clinical diagnoses but on mothers’ reports, we cannot exclude the possibility of biased maternal reporting based on pregnancy complications/ offspring illness and increased attention. However, there are acknowledged associations between several pregnancy complications and clinically diagnosed NDDs [77, 78], many of which are believed to be causal, e.g., severe prematurity [79–81]. In addition, the data on organic food is collected prospectively, before birth, so complications with birth or illness in child could not influence the exposure. Thus, we do not believe that biased reporting will completely explain the associations.

We also included symptoms of anxiety and depression in mothers and fathers as covariates, with no impact on the results for offspring ASD symptoms as outcome. In relation to offspring ADHD symptoms as an outcome, we also included maternal symptoms of ADHD as an additional covariate with non-significant results (no measure of parental ASD symptoms was available).

We used within-sibling analyses to evaluate unmeasured familial confounding, i.e., shared family environment and genetics. Again, estimates weakened or became non-significant for both ADHD and “high” ASD symptom scores. Taken together, these findings suggest that the observed associations may largely be explained by unmeasured confounding factors, and not by organic food intake per se. Such confounding could be due to genetic or family-environmental factors shared by siblings.

However, we cannot be certain about this interpretation of the findings. Firstly, design limitation such as data collection in fathers and mothers at different time points and use of brief scales of anxiety, depression, and ADHD make the findings less conclusive. Furthermore, the lower number of participants in the sibling analyses as compared to the main analyses yielded lower statistical power to detect effects and also much higher variance [82]. Although there is general agreement that sibling comparison analyses require very large samples sizes, there is no clear consensus regarding exact number of participants, as this also depends on other study characteristics [83]. The sample sizes in the sibling analyses are considerably smaller than in the ADHD and ASD study samples, 13.6% of the ADHD and 12.7%/19.4% of the ASD “low” versus “high” organic study samples, respectively. As shown in Figs. 3 and 4, the confidence intervals in the sibling comparison analyses are much wider than in the main analyses, indicating that the sample sizes are less optimal for such analyses. However, the distribution between measured characteristics (as those presented in Table 1) were quite similar between exposure-discordant and exposure-concordant siblings and between discordant versus total sample, indicating that information from the discordant siblings with caution may be generalized to the whole sample. Also, while sibling comparisons have strong benefits, they may also introduce biases in the findings [82, 84, 85]. They may for instance increase confounding by unshared factors between siblings, such as from sibling-specific genetic differences or unmeasured systematically discordant exposures. Altogether, care must be taken when interpreting the results from the sibling analyses. However, in our study, the results from the sibling analyses were quite similar to the main model.

We also considered cross-over effects, i.e., dietary change from one pregnancy to the next. Cross-over effects are however unlikely, as participants were recruited within 8 consecutive years, and the mean duration between the pregnancies was 2.6 years, both in the exposure concordant and discordant sibling pairs. As symptoms of ASD and ADHD usually become evident some years after birth, the mother would not have enough time to change her dietary habits from the previous to the current pregnancy.

The MoBa FFQ has been shown to be valid on intakes of energy, nutrients, and food among pregnancy women [35]. Although the specific questions on organic food consumption have not been validated, this information can be considered a robust indicator of the main categories of organic food [38]. There are no specific details available regarding the exact amount of food eaten nor the amount of nutrients or toxic components in the different food categories. Thus, we were not able to study if women who ate organic food had different intake of favorable nutrients or other substances compared to women who did not use organic food. The dietary measures were crude and may not identify large enough contrast to detect differences when comparing with non-organic food. However, other MoBa studies have detected associations between frequent organic consumption in pregnancy and health outcomes [29, 30].

One additional limitation was that information on organic food intake in the child itself was not available and therefore, possible confounding and intermediate effects [17] of childhood diet on the observed associations could not be assessed. Furthermore, selection bias due to self-selection to participate and loss-to-follow up is a concern in cohort studies like MoBa [33, 86]. Compared to participants lost-to-follow-up, the mothers who remained in the cohort were on average older, higher educated, had lower BMI and smoked less [87]. While selection bias could lead to both under-and overestimation of exposure-outcome associations in MoBa [33], a previous MoBa study focusing on ASD diagnosis as outcome found that self-selection only had a minor impact on the association estimates [88]. Furthermore, we did not use inverse probability weighting (IPW), a method used to decrease bias due to loss-for-follow up. Previous MoBa studies with similar design as the present study have not shown signs of severe selection bias when using IPW [14, 17, 88, 89]. Taking these limitations into account, the results might, with caution, be generalized to Western industrialized populations similar to the MoBa’s source population. However, the generalizability to populations with limited access to healthy food can be questioned. Finally, as this is an observational study, it is not possible to identify causal relationships with certainty. Although we adjusted for a number of covariates and used within-sibling analyses to evaluate unmeasured familial confounding, there could still be residual confounding not accounted for.

## Conclusions

The observed associations between frequent consumption of organic food during pregnancy and symptoms of ADHD and ASD in the offspring were small or not significant. The results do not indicate any clinically significant protective or harmful effects of eating organic food during pregnancy on symptoms of ADHD and ASD in the offspring. Based on these findings, we do not recommend any specific advice regarding intake of organic food during pregnancy.

## Supplementary Information

Additional file 1: Overview number of missing items and values imputed in exposure and outcome variables. Additional file 2: Method on categorization of exposures and outcomes, and related statistics. Additional file 3: Tab. S1. Further sample characteristics of mother-child pairs. Additional file 4: Tab. S2. Sample characteristics of mother-child pairs with categorical outcomes. Additional file 5: Tab. S3. Number of participants (%) reporting scoring often/usually consumed food from the specific different food categories. Additional file 6: Fig. S1. “High” versus “low” ADHD symptoms in offspring by maternal organic food intake (categorized) in pregnancy. Additional file 7: Fig. S2. Symptoms of ADHD and ASD in offspring by maternal organic food intake (continuous sum score) – including data from fathers. Additional file 8: Fig. S3. Symptoms of ADHD and ASD in offspring by maternal organic food intake (continuous sum score) – stratified by sex. Additional file 9: Fig. S4. Symptoms of ADHD and ASD in offspring by organic food groups. Additional file 10: Fig. S5. “High” versus“low” ASD symptoms in offspring by maternal organic food intake (categorized) in pregnancy.

## Data Availability

Data from the Norwegian Mother, Father and Child Cohort Study and the Medical Birth Registry of Norway used in this study are managed by the national health register holders in Norway (Norwegian Institute of public health) and can be made available to researchers, provided approval from the Regional Committees for Medical and Health Research Ethics (REC), compliance with the EU General Data Protection Regulation (GDPR) and approval from the data owners. The consent given by the participants does not open for storage of data on an individual level in repositories or journals. Researchers who want access to data sets for replication should apply through helsedata.no. Access to data sets requires approval from The Regional Committee for Medical and Health Research Ethics in Norway and an agreement with MoBa.

## Abbreviations

ADHD: Attention deficit/hyperactivity disorder
ASD: Autism spectrum disorder
ASRS-6: The Adult ADHD Self-Report Scale Screener
BMI: Body mass index
FFQ: Food frequency questionnaire
MoBa: The Norwegian Mother, Father and Child Cohort Study
MBRN: The Medical Birth Registry of Norway
NDD: Neurodevelopmental disorders
RS-DBD: Parent/Teacher Rating Scale for Disruptive Behaviour Disorders
SCL-8: The Hopkins Symptom Checklist-25
SCQ: The Social Communication Questionnaire
