# Supplementary material for: Organic food consumption during pregnancy and symptoms of neurodevelopmental disorders at 8 years of age in the offspring: the Norwegian Mother, Father and Child Cohort Study (MoBa)
Source: BMC Med. 2024 Oct 21;22:482. doi: 10.1186/s12916-024-03685-5 (PMC11492991; doi:10.1186/s12916-024-03685-5)
Supplement: Supplementary file 1 — Additional file 1: Overview number of missing items and values imputed in exposure and outcome variables. Additional file 2: Method on categorization of exposures and outcomes, and related statistics. Additional file 3: Tab. S1. Further sample characteristics of mother-child pairs. Additional file 4: Tab. S2. Sample characteristics of mother-child pairs with categorical outcomes. Additional file 5: Tab. S3. Number of participants (%) reporting scoring often/usually consumed food from the specific different food categories. Additional file 6: Fig. S1. “High” versus “low” ADHD symptoms in offspring by maternal organic food intake (categorized) in pregnancy. Additional file 7: Fig. S2. Symptoms of ADHD and ASD in offspring by maternal organic food intake (continuous sum score) – including data from fathers. Additional file 8: Fig. S3. Symptoms of ADHD and ASD in offspring by maternal organic food intake (continuous sum score) – stratified by sex. Additional file 9: Fig. S4. Symptoms of ADHD and ASD in offspring by organic food groups. Additional file 10: Fig. S5. “High” versus“low” ASD symptoms in offspring by maternal organic food intake (categorized) in pregnancy. [file 12916_2024_3685_MOESM1_ESM.pdf]

ADDITIONAL FILES.

TITLE:

Organic food consumption during pregnancy and symptoms of neurodevelopmental disorders at 8 years of age in the offspring: The Norwegian Mother, Father and Child Cohort Study (MoBa)

AUTHORS:

Johanne T. Instanes[1], Berit S. Solberg[2,3], Liv G. Kvalvik[1], Kari Klungsøyr[1,4], Maj-Britt R. Posserud[5,6,7], Catharina A Hartman[8], Jan Haavik[9]

Affiliations:

[1]Department of Global Public Health and Primary Care, University of Bergen, Norway
[2]Department of Biomedicine, University of Bergen, Bergen, Norway
[3]Child- and adolescent psychiatric outpatient unit, Hospital Betanien, Bergen, Norway
[4]Division of Mental and Physical Health, Norwegian Institute of Public Health, Bergen, Norway
[5]Department of Clinical Medicine, University of Bergen, Bergen, Norway
[6]Division of Psychiatry, Haukeland University Hospital, Bergen, Norway
[7]Gillberg Neuropsychiatry Centre, Institute of Neuroscience and Physiology, the Sahlgrenska Academy, University of Gothenburg, Gothenburg, Sweden.
[8]Department of Psychiatry, Interdisciplinary Center Psychopathology and Emotion Regulation, University Medical Center Groningen, University of Groningen, Groningen, The Netherlands.
[9]Bergen Center for Brain Plasticity, Division of Psychiatry, Haukeland University Hospital, Bergen, Norway

Corresponding author

Johanne Telnes Instanes, Johanne.instanes@uib.no , PO.box 7804, N-5020 Bergen, Norway

Additional files

Additional file 1: Overview number of missing items and values imputed in exposure and outcome variables

Additional file 2: Method on categorization of exposures and outcomes, and related statistics.

Additional file 3: Tab. S1. Further sample characteristics of mother-child pairs

Additional file 4: Tab. S2. Sample characteristics of mother-child pairs with categorical outcomes

Additional file 5: Tab. S3. Number of participants (%) reporting scoring often/usually consumed food from the specific different food categories

Additional file 6: Fig. S1. "High" versus "low" ADHD symptoms in offspring by maternal organic food intake (categorized) in pregnancy

Additional file 7: Fig. S2. Symptoms of ADHD and ASD in offspring by maternal organic food intake (continuous sum score) – including data from fathers

Additional file 8: Fig. S3. Symptoms of ADHD and ASD in offspring by maternal organic food intake (continuous sum score) – stratified by sex

Additional file 9: Fig. S4. Symptoms of ADHD and ASD in offspring by organic food groups

Additional file 10: Fig. S5. "High" versus "low" ASD symptoms in offspring by maternal organic food intake (categorized) in pregnancy.

ADDITIONAL FILE 1

Overview number of missing items and values imputed in exposure and outcome variables.

Exposure: Frequency of organic food intake during pregnancy
- information based on 6 items from the MoBa food frequency questionnaire (FFQ).

Missing items	0	1	2	3	4	5	(6)
n<br>(%)	39,564<br>(97.2)	554<br>(1.4)	107<br>(0.3)	108<br>(0.3)	150<br>(0.4)	224<br>(0.6)	(Not included in study population)

Study population N=40,707.
Of a total of 244242 values, 2812 (1.15%) were imputed by mean imputation.
In an alternative model, all missing items were scored as 0, with basically the same results as compared with mean imputation model.

Outcome: Symptoms of ADHD (Attention-deficit/hyperactivity disorder)-
Information based on 18 items from the Parent/Teacher Rating Scale for Disruptive Behaviour Disorders

Continuous outcome:
Children missing nine or more items were excluded (n=121(0.3%)). If eight or less items were missing (n=1,347(3.3%)), missing values were imputed by mean imputation. Of a total of 730548 values, 1808 (0.25%) were imputed by mean imputation.

Categorical outcome:
If more than four out of nine items in one or both of the two symptom categories inattentive and hyperactive/impulsive were missing, the cases were defined as missing and omitted from further analyses (n=136(0.3%)).

Outcome: Symptoms of ASD (autism spectrum disorder)
Information based on The Social Communication Questionnaire (SCQ) (40 items)
Total N= 40,707.
Excluded due to no phrase speech: n=278 (0.7%)
After first exclusion n=40,429.
Excluded due to missing ≥19 of the remaining 39 items: n=310(0.8%)
Excluded due to error in filling out the questionnaire: n=2
After second exclusion n=40,117
Remaining missing items imputed by mean imputation: 10793(0.7%) of total 1564641 values.
Of n=5208(13.0%) cases with imputed values, n=3105(7.7%) had one missing value only.

ADDITIONAL FILE 2

Method on categorization of exposures and outcomes and related statistics

Categorizations of exposures and outcomes:

<u>Exposure:</u>

The sumscore of organic food (0-18) was categorized as follows: 0 (no intake organic food), 1-3;4-6;7-10;11-14 and 15-18.

Due to the sumscore distribution, , the sumscore values are not equally distributed within the categories. The categories with the highest organic sumscores are more broadly categorized compared to the lower scorers, as to have sufficient of participants to be included in regression analyses.

<u>Outcome:</u>

ADHD symptoms

Further, scores were categorized as “high” versus "low”. A “high” ADHD symptom score was defined as ≥6 items scored as ’often’ or ’very often’ in the inattentive category and/or in the hyperactive/impulsive category. The remaining sample was categorized as “low”. Similar categorization has been used previously in a MoBa study, and the ≥6 cut -off resembles the diagnostic symptom criteria for ADHD when the data was collected; that 6 or more out of 9 symptoms in the inattentive category and/or in the hyperactive/impulsive should be present to diagnose combined ADHD or a subtype, accordingly (1). Questionnaires missing ≥4 answers in one or both categories were excluded (n=136(0.3%)).

ASD symptoms

Second, scores were categorized using a cutoff score of 15 commonly used to indicate the likelihood of an individual having ASD or not (2). “High” versus “low” ASD symptom scores were defined as scoring ≥15 and ≤14, respectively.

Statistics:

Relative risks (RR) with 95% CI were estimated using log-binomial regression models for categorical exposure and outcomes.

The covariates were treated as continuous when the relationship between the covariates and outcomes were linear. With non-linear relationships, the covariates were categorized or spline modelling used when applicable to not loose statistical power.

The following covariates were included: birth year; birth season (ADHD only) (January– March, April–June, July–September, October–December); maternal age at delivery (restricted cubic spline with 3 knots); maternal educational level (low, medium and high (less than high school, high school or college/university)); parity(nulliparous, 1 previous pregnancy or ≥ 2 previous pregnancies); pre-pregnancy body-mass index (BMI) calculated from self-reported height and weight (normal weight, overweight or obesity (< 18.5, 18.5–24.9, 25–29.9 and ≥30 kg/m2); smoking during pregnancy (yes/no); alcohol intake during pregnancy (yes/no); maternal total energy intake (kcal/day); maternal fiber consumption (gram/day); maternal symptoms of anxiety and depression (score1-4) and maternal symptoms of ADHD (score 0-24).

Details on maternal symptoms of anxiety and depression are found in manuscript, section "covariates".

1. Kvalvik LG, Klungsoyr K, Igland J, Caspersen IH, Brantsaeter AL, Solberg BS, et al. Association of sweetened carbonated beverage consumption during pregnancy and ADHD symptoms in the offspring: a study from the Norwegian Mother, Father and Child Cohort Study (MoBa). Eur J Nutr. 2022;61(4):2153-66.

2. Rutter M, Bailey A, Lord C. The social communication questionnaire: Manual. Los Angeles, CA: Western Psychological Services; 2003.

ADDITIONAL FILE 3: **Tab. S1.**

Further sample characteristics of mother-child pairs

**Study sample** [a]	**ADHD study sample** [b] n(%)	**ASD study sample** [c] n(%)	
		"Low" Organic sumscore (0-5)	"High" Organic sumscore (6-18)
Mother-child pairs	40 586 (100)	34 421 (100)	5 696 (100)
Prepregnancy body mass index (BMI)			
Mean score (SD) [d]	23.9 (4.1)	24.0 (2.1)	23.4 (3.9)
Birth season			
Born January-March	10 481 (25.8)	8 901 (25.9)	1 470 (25.8)
Born April-June	10 297 (25.4)	8 711 (25.3)	1 475 (25.9)
Born July-September	10 765 (26.5)	9 155 (26.6)	1 474 (25.9)
Born October-December	9 043 (22.3)	7 654 (22.2)	1 277 (22.4)
Maternal energy intake (kcal/day)			
Mean score (SD)	2 305 (627)	2290 (615)	2393 (690)
Maternal fiber intake (grams/day)			
Mean score (SD)	31.1 (10.5)	30.6 (10.2)	34.0 (12.1)
Alcohol during pregnancy [e]			
No	35 981 (88.7)	30 576 (88.8)	4 985 (87.5)
Yes	4 605 (11.4)	3 845 (11.2)	711 (12.5)
Smoking during pregnancy			
No	34 616 (85.3)	29 411 (85.4)	4 809 (84.4)
Yes	5 036 (12.4)	4 257 (12.4)	713 (12.5)
Missing information	934 (2.3)	753 (2.2)	174 (3.1)
Maternal symptoms of ADHD [f]			
Mean score (SD)	6.4 (3.4)	6.4 (3.3)	6.7 (3.5)
Missing	7 166 (17.7)	5 942(17.3)	1 122 (19.7)
Maternal symptoms of anxiety and depression [g]			
Mean score (SD)	1.2 (0.3)	1.2 (0.3)	1.3 (0.4)
Missing	822 (2.0)	655	155 (1.9)

[a] Based on the Norwegian Mother, Father and Child Cohort Study (MoBa).
[b] Attention deficit/hyperactivity disorder (ADHD)
[c] Autism spectrum disorder (ASD). ASD study sample stratified into two; organic sumscore 0-5 ("low" organic sumscore; 0-5) and 6-18 ("high" organic sumscore; 6-18)
[d] SD = Standard deviation
[e] Missing categorized as "no"
[f] Symptoms of maternal anxiety and depression the last two weeks assessed by eight items from the Hopkins Symptom Checklist-25 (SCL-8) around gestational week 30, mean score with the range of 1-4
[g] Symptoms of ADHD in mothers during the last six months from the Adult ADHD Self-Report Scale Screener (ASRS-6) when the child was three years, summary score with the range 0-24

ADDITIONAL FILE 4 **Tab. S2.**

Sample characteristics of mother-child pairs with categorical outcomes

**Study sample[a]**	**ADHD symptom score[b]**		**ASD symptom score[c]**	
	Low (n,%)	High (n,%)	Low (n,%)	High (n,%)
Mother-child pairs	39 008 (96.2)	1 563 (3.9)	39 961 (99.6)	156 (0.4)
Birth year				
2002	1 920 (4.9)	103 (6.6)	1 990 (5.0)	15 (9.6)
2003	5 553 (14.2)	227(14.5)	5 675 (14.2)	28 (18.0)
2004	5 406 (13.9)	216 (13.8)	5 563 (13.9)	22 (14.1)
2005	6 228 (16.0)	237 (15.2)	6 355 (15.9)	22 (14.1)
2006	7008 (18.0	254 (16.3)	7 156 (17.9)	22 (14.7)
2007	6423 (16.5)	250 (16.0)	6 551 (16.4)	23 (14.7
2008	5124 (13.1)	223 (14.3)	5 284 (13.2)	22 (14.0)
2009	1346(3.5)	53 (3.4)	1 387 (3.5)	2 (1.3)
Maternal age at delivery (years)				
<17-19	165 (0.4)	18 (1.2)	174 (0.4)	3 (1.9)
20-24 years	2 863 (7.3)	175 (11.2)	2 975 (7.4)	26 (16.7)
25-29 years	12 445 (31.9)	538 (34.4)	12 813 (32.1)	46 (29.5)
30-34 years	16 022 (41.1)	560 (35.8)	16 351 (40.9)	52 (33.3)
35-39 years	6 606 (16.9)	245 15.7)	6 742 (16.9)	24 (15.4)
40 years or older	907 (2.3)	27 (1.7)	906 (2.3)	5 (3.2)
Maternal education [d]				
Less than high school	590 (1.5)	55 (3.5)	614 (1.5)	10 (6.4)
High school	10 636 (27.3)	602 (38.5)	11 008 (27.6)	74 (47.4)
≥4 years college/university	26 966 (69.1)	867 (55.5)	27 500 (68.8)	69 (44.2)
Missing	816 (2.1)	39 (2.5)	839 (2.1)	3 (1.9)
Gestational length				
<34	443 (1.1)	39 (2.5)	470 (1.2)	6 (3.8)
≥34	38 565 (98.9)	1 524 (97.5)	39 491 (98.8)	150 (96.2)
Maternal symptoms of depression and anxiety [g]				
Mean score (SD)	1.2 (0.3)	1.4 (0.5)	6.4 (3.3)	8.2 (3.8)
Missing	793 (2.0)	7 (5.2)	802 (2.0)	8 (5.1)

[a] Based on the Norwegian Mother, Father and Child Cohort Study (MoBa).

[b] ADHD (attention-deficit/hyperactivity disorder) study sample. Offspring symptoms of ADHD at 8 years of age categorized into low and high ADHD symptom scores based on information from the Parent/Teacher Rating Scale for Disruptive Behaviour Disorders

[c] Based on the ASD (autism spectrum disorder) study sample. Offspring symptoms of ASD screened at 8 years of age categorized into low and high symptom scores based on information from the Social Communication Questionnaire(0-39) with a cutoff score of 15.

ADDITIONAL FILE 5: **Tab. S3.**

Number of participants (%) reporting scoring often/usually consumed food from the specific organic food group

Organic food group[a]	Milk/dairy	Bread/ cereals	Eggs	Vegetables	Fruit	Meat
Often/usually n(%) [b]	2 831(7.0)	2 273(5.6)	4 137(10.2)	3 001(7.4)	2 501(6.1)	1 333 (3.3)

[a] Missing ≤0.03% for each different food category
[b] Often/usually consumption of the specific organic food group, as scored on the six questions on organic food on the MoBa food frequency questionnaire

ADDITIONAL FILE 6: Fig. S1

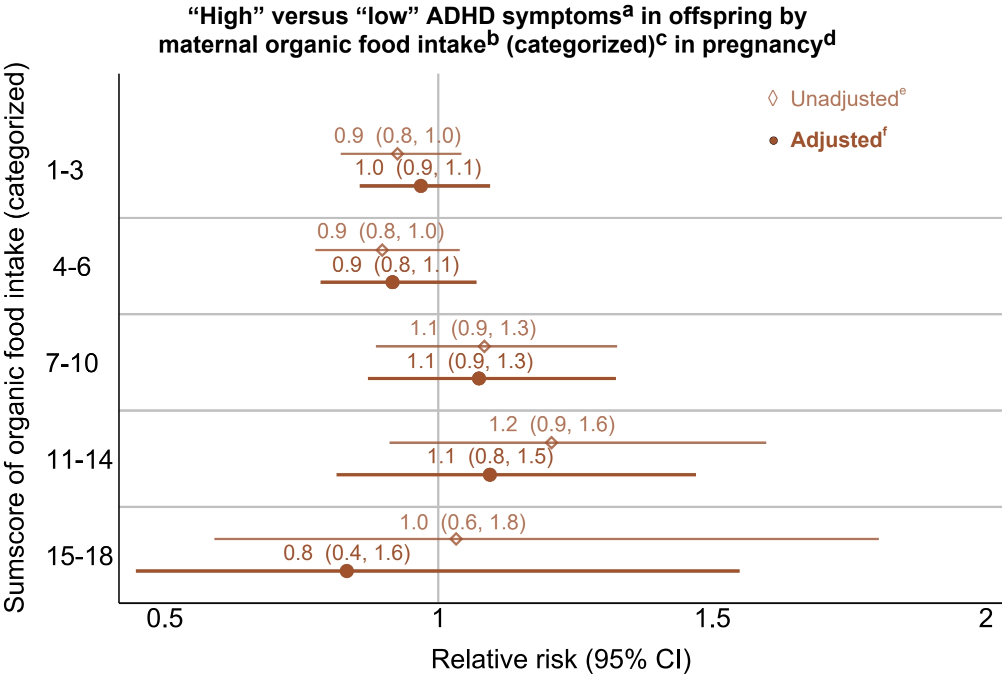

[a] Offspring symptoms of attention-deficit/hyperactivity disorder (ADHD) at 8 years of age categorized into low and high ADHD symptom scores based on information from the Parent/Teacher Rating Scale for Disruptive Behaviour Disorders (RS-DBD).

[b] Continuous sum score of organic food intake (0-18) represent the frequency of maternal organic food intake during pregnancy; from 0 representing no/seldom intake to 18 representing frequent intake

[c] Sum score of organic food intake (0-18) categorized as follows: 0;1-3;4-6;7-10;11-14;15-18

[d] Data from the Norwegian Mother, Father and Child Cohort Study (MoBa)

[e] Unadjusted log-binominal regression model (n=40 571)

[f] Adjusted log-binominal model (n=37 804 complete cases). Adjusted for birth year; birth season; maternal age at delivery; maternal educational level; parity; pre-pregnancy body mass index (BMI); maternal smoking, alcohol, energy and fiber intake during pregnancy; maternal symptoms of depression and anxiety in pregnancy measured around gestational week 30

ADDITIONAL FILE 7: Fig. S2

Symptoms of ADHD[a] and ASD[b] in offspring by maternal organic food intake (continuous sum score)[c] - including data from fathers[d]

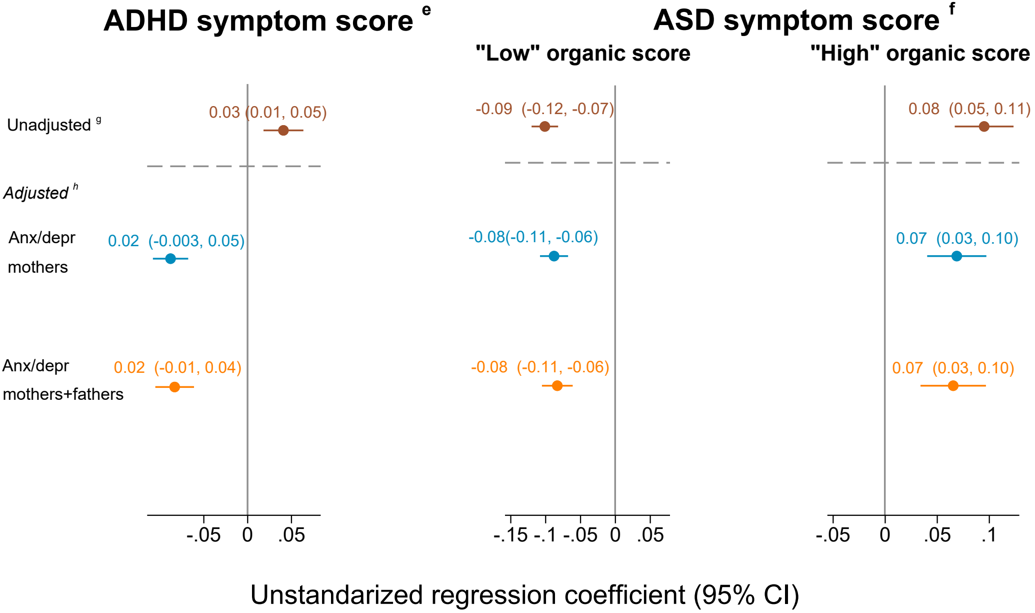

[a] Offspring symptoms of attention-deficit/hyperactivity disorder (ADHD) screened at 8 years of age by the Parent/Teacher Rating Scale for Disruptive Behaviour Disorders (RS-DBD)

[b] Offspring symptoms of autism spectrum disorder (ASD) screened at 8 years of age by the Social Communication Questionnaire (SCQ)

[c] Continuous sum score of organic food intake (0-18) represent the frequency of maternal organic food intake during pregnancy; from 0 representing no/seldom intake to 18 representing frequent intake

[d] Data from the Norwegian Mother, Father and Child Cohort Study (MoBa), a subsample including questionnaire data from fathers in addition to mothers.

[e] Continuous ADHD symptom score 0-54 based on information from RS-DBD

[f] Continuous ASD symptom score 0-39 based on information from SCQ. The association between the continuous ASD symptom score and the sumscore of organic food intake (total range 0-18) was non-linear. The linear regression analysis were thus stratified into two: “low” organic sumscore(0-5) and high organic sumscore”(6-18)

[g] Unadjusted linear regression; subsample including mothers and fathers with questionnaire data on own symptoms of anxiety and depression, n = 32 806 (ADHD study sample) and n = 32 445 (ASD study sample)

[h] Adjusted linear regression. Pay attention to different scaling on the x-axes for ADHD and ASD. Adjusted for birth year; birth season (for ADHD only); maternal age at delivery; maternal educational level; parity; pre-pregnancy body mass index (BMI); maternal smoking, alcohol, energy and fiber intake during pregnancy; maternal symptoms of depression and anxiety in pregnancy measured around gestational week 30 and in fathers around week 15. Data on fathers only used in adjustment model: “anx/depr mothers+fathers”. Total n for complete cases varied between 31 297 and 30 957 for the ADHD and ASD study sample, respectively.

ADDITIONAL FILE 8: Fig. S3

Symptoms of ADHD[a] and ASD[b] in offspring by maternal organic food intake (continuous sum score)[c,d] – stratified by sex

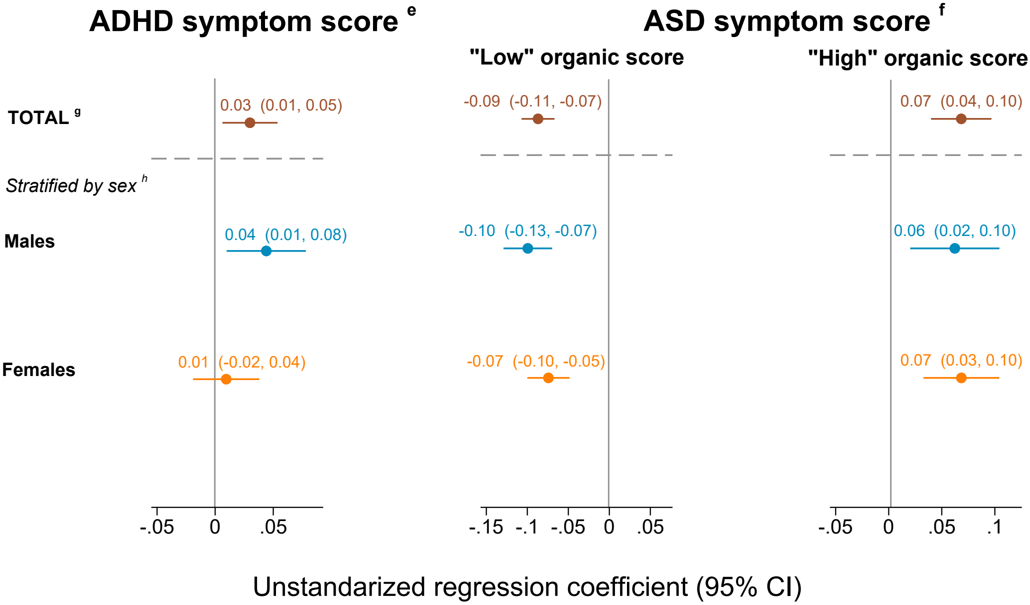

[a] Offspring symptoms of attention-deficit/hyperactivity disorder (ADHD) screened at 8 years of age by the Parent/Teacher Rating Scale for Disruptive Behaviour Disorders (RS-DBD)

[b] Offspring symptoms of autism spectrum disorder (ASD) screened at 8 years of age by the Social Communication Questionnaire (SCQ)

[c] Continuous sum score of organic food intake (0-18) represent the frequency of maternal organic food intake during pregnancy; from 0 representing no/seldom intake to 18 representing frequent intake

[d] Data from the Norwegian Mother, Father and Child Cohort Study (MoBa)[e] Continuous ADHD symptom score 0-54 based on information from RS-DBD

[e] Continuous ADHD symptom score 0-54 based on information from RS-DBD

[f] Continuous ASD symptom score 0-39 based on information from SCQ. The association between the continuous ASD symptom score and the sumscore of organic food intake (total range 0-18) was non-linear. The linear regression analysis were thus stratified into two: "low" organic sumscore(0-5) and high organic sumscore"(6-18)

[g] Total; i.e. not stratified. Adjusted linear regression. Pay attention to different scaling on the x-axes for ADHD and ASD. Adjusted for birth year; birth season (for ADHD only); maternal age at delivery; maternal educational level; parity; pre-pregnancy body mass index (BMI); maternal smoking, alcohol, energy and fiber intake during pregnancy, maternal symptoms of depression and anxiety in pregnancy measured around gestational week 30. Complete cases: n= 37 822 (ADHD study sample) and n= 37 394 (ASD study sample)

[h] Results stratified by offspring sex.

ADDITIONAL FILE 9: Fig. S5

Symptoms of ADHD[a] and ASD[b] in offspring by organic food groups[c,d]

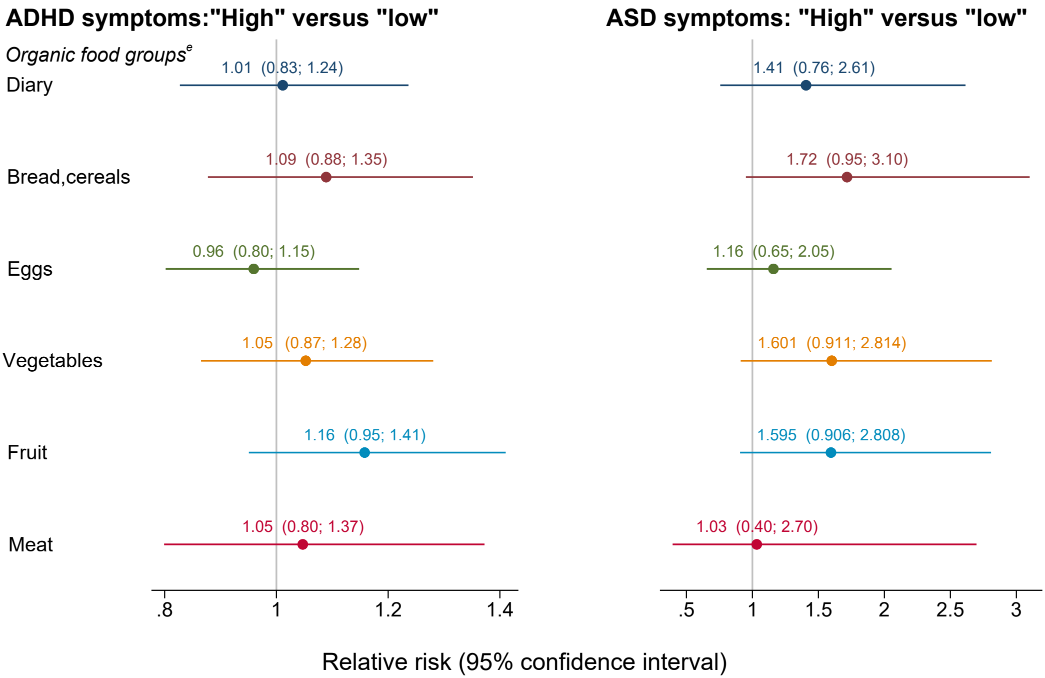

[a] Offspring symptoms of attention-deficit/hyperactivity disorder (ADHD) at 8 years of age categorized into low and high ADHD symptom scores based on information from the Parent/Teacher Rating Scale for Disruptive Behaviour Disorders (RS-DBD)

[b] Offspring symptoms of autism spectrum disorder (ASD) screened at 8 years of age; categorized into low and high symptom scores based on information from the Social Communication Questionnaire (SCQ) (0-39), with a cutoff score of 15

[c] For each of the six organic food groups, often/usually consumption of the food group (for instance often/usually eat organic eggs) was compared with never eating organic food

[d] Data from the Norwegian Mother, Father and Child Cohort Study (MoBa)

[e] Adjusted log-binominal model . Pay attention to different scaling on the x-axes for ADHD and ASD. Adjusted for birth year; birth season (for ADHD only); maternal age at delivery; maternal educational level; parity; pre-pregnancy body mass index (BMI); maternal smoking, alcohol, energy and fiber intake during pregnancy, maternal symptoms of depression and anxiety in pregnancy measured around gestational week 30. Total n varied between 19 409 and 22 259

ADDITIONAL FILE 10: Fig. S5

"High" versus "low" ASD symptoms[a] in offspring by maternal organic food intake[b] (categorized)[c] in pregnancy[d]

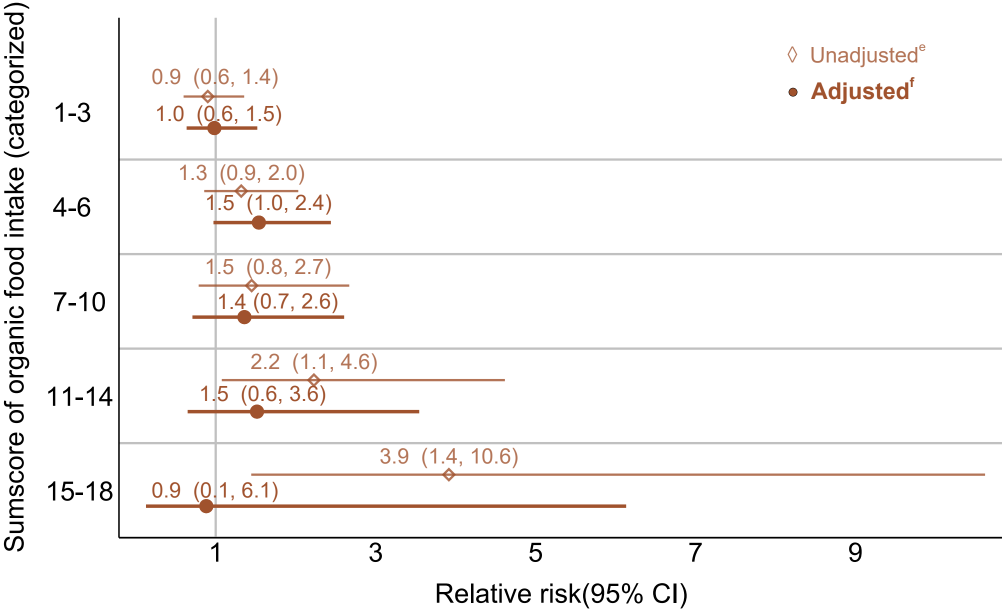

[a] Offspring symptoms of autism spectrum disorder (ASD) at 8 years of age categorized into low and high ASD symptom scores based on information from the Social Communication Questionnaire (SCQ)(0-39), cut-off score

[b] Continuous sum score of organic food intake (0-18) represent the frequency of maternal organic food intake during pregnancy; from 0 representing no/seldom intake to 18 representing frequent intake

[c] Sum score of organic food intake (0-18) categorized as follows: 0;1-3;4-6;7-10;11-14;15-18

[d] Data from the Norwegian Mother, Father and Child Cohort Study (MoBa)

[e] Unadjusted log-binominal regression model (n=40 117)

[f] Adjusted log-binominal model (n=37 409 complete cases). Adjusted for birth year; maternal age at delivery; maternal educational level; parity; pre-pregnancy body mass index (BMI); maternal smoking, alcohol, energy and fiber intake during pregnancy, maternal symptoms of depression and anxiety in pregnancy measured around gestational week 30
